# Rituximab maintenance therapy of follicular lymphoma in clinical practice

**DOI:** 10.1002/cam4.1549

**Published:** 2018-05-15

**Authors:** Ulrich Dührsen, Stefanie Broszeit‐Luft, Annette Dieing, Andreas Lück, Piotr Porowski, Marcel Reiser, Ulrike Schwinger, Sandra Klawitter, Katja Krumm, Kathleen Jentsch‐Ullrich

**Affiliations:** ^1^ Department of Hematology University Hospital Essen Essen Germany; ^2^ Oncological Practice Lehrte Germany; ^3^ Vivantes Medical Care Center at Urban Berlin Germany; ^4^ Center for Oncology and Urology at Rostock Rostock Germany; ^5^ Oncological Practice Heilbronn Germany; ^6^ Practice for Internal Oncology and Hematology Cologne Germany; ^7^ Oncological Practice Stuttgart Germany; ^8^ Roche Pharma AG Grenzach‐Wyhlen Germany; ^9^ Practice for Hematology and Oncology Magdeburg Germany

**Keywords:** chemoimmunotherapy, follicular lymphoma, observational study, rituximab

## Abstract

Standard of care for patients with symptomatic, advanced‐stage follicular lymphoma (FL) is rituximab‐containing chemoimmunotherapy followed by rituximab maintenance. This prospective, multicenter, noninterventional study analyzed how efficacy and safety data from randomized controlled trials translate into clinical practice in Germany. Both treatment‐naïve and relapsed/refractory patients with FL, who responded to rituximab‐containing induction and were scheduled for rituximab maintenance, were observed for 24 months. Effectiveness was measured by response and Kaplan‐Meier survival analysis. In addition, treatment patterns of induction and maintenance, as well as adverse events, were documented. The evaluable study population consisted of 310 first‐line patients and 173 relapsed/refractory patients, including 116 patients with initial Ann‐Arbor stage I/II and 20 patients with FL grade 3B. Regarding first‐line induction, a shift from R‐CHOP (rituximab, cyclophosphamide, doxorubicin, vincristine, prednisone) to R‐bendamustine was observed over time, as well as a decline in radiotherapy. 2‐year progression‐free survival rates were 88.3% (95% confidence interval [CI] 84.0‐92.6) for first‐line patients and 76.0% (95% CI: 68.8‐83.3) for relapsed/refractory patients. Conversion from partial to complete remission (PR, CR) occurred in 53.4% of analyzed first‐line patients with PR, resulting in 69.4% CRs at study end (relapsed/refractory: conversion in 42.9%, final CRs 57.9%). Safety results were consistent with the known safety profile of rituximab in this setting. Both treatment‐naïve and relapsed/refractory patients with FL show favorable 2‐year PFS rates and improvements in the remission status with postinduction rituximab monotherapy as maintenance and consolidation therapy.

## 1 INTRODUCTION

Follicular lymphoma (FL) is the second most common type of non‐Hodgkin's lymphoma, increasing in incidence especially in Western countries.^1^, ^2^ Based on the proportion of centrocytes and centroblasts, the World Health Organization (WHO) classification distinguishes FL grades 1, 2, 3A, and 3B.^3^, ^4^ Whereas FL grades 1, 2, and 3A are indolent, grade 3B is generally considered as an aggressive lymphoma and treated with curative intent according to the recommendations for diffuse large B‐cell lymphoma.^4^, ^5^ Approximately 89% of patients with indolent FL are diagnosed in Ann‐Arbor stages III or IV, which are not curable with conventional therapy.^6^

The advent of the anti‐CD20 antibody rituximab significantly improved the treatment options for FL. For remission induction, rituximab is most frequently combined with chemotherapy, such as CHOP (cyclophosphamide, doxorubicin, vincristine, prednisone),^7^ bendamustine,^8^ or CVP (cyclophosphamide, vincristine, prednisone).^9^

Maintenance therapy with rituximab is frequently followed by a prolonged remission period, both after first‐line^10^, ^11^, ^12^ and after salvage induction therapy.^13^, ^14^ In the largest randomized controlled trial in treatment‐naïve patients with FL, the phase 3 Primary Rituximab and Maintenance (PRIMA) study, 2 years of rituximab maintenance following rituximab‐containing chemotherapy (CVP, CHOP, or FCM [fludarabine, cyclophosphamide, mitoxantrone]) was assessed in 1217 patients. Maintenance therapy achieved complete remission (CR) in 72% of the patients compared with 52.2% in the observation arm (*P* = .0001),^12^ and 6‐year progression‐free survival (PFS) rates were 59.2% vs 42.7% (*P* < .0001; hazard ratio [HR] 0.58); however, 6‐year overall survival (OS) estimates were similar.^15^

The largest phase 3 trial in relapsed/refractory FL was conducted by the European Organisation for Research and Treatment of Cancer (EORTC 20981) and investigated rituximab in remission induction and maintenance treatment of 465 patients.^13^, ^16^ Patients responding to induction with either CHOP or R‐CHOP (rituximab plus CHOP) were randomly assigned to rituximab maintenance or observation. Maintenance therapy significantly prolonged PFS compared with observation (median PFS, 3.7 years vs 1.3 years; *P* < .001; HR 0.55), following either CHOP or R‐CHOP. While there was no statistically significant difference in OS in this trial,^13^ a recent meta‐analysis came to the conclusion that rituximab maintenance does improve OS of relapsed/refractory patients with FL.^17^

Rituximab plus chemotherapy followed by rituximab maintenance has thus been established as a widely accepted standard of care for patients with symptomatic, advanced‐stage FL.^5^, ^18^ To assess whether the results from randomized controlled studies translate into clinical practice, the present noninterventional study investigated the effectiveness, safety, and treatment patterns of rituximab maintenance for 24 months after rituximab‐containing induction therapy in patients with FL under routine conditions.

## 2 MATERIALS AND METHODS

This prospective, noninterventional study (ClinicalTrials.gov Identifier: NCT02536664) was conducted between August 2009 and June 2014 at 138 centers in Germany, including hospitals, outpatient clinics, and office‐based practices.

This study was conducted in accordance with Good Pharmacoepidemiological Practice (GPP). The study protocol was approved by the Medical Faculty ethics committee at the University of Duisburg‐Essen. Patients were required to provide written informed consent.

Adult patients with previously untreated (cohort 1), or relapsed or refractory CD20‐positive FL (cohort 2), who achieved CR or partial remission (PR) following rituximab‐containing induction therapy and were scheduled to receive rituximab maintenance therapy, were eligible for the study.

Individual treatment schedules, dose and frequency of administration of intravenous rituximab (MabThera^®^, Roche, Basel, Switzerland), diagnostic and therapeutic interventions, frequency of visits, and other treatment decisions were made by the physician prior to and independent of enrollment.

Data were documented at prespecified time points during the planned observation time of 24 months. Adverse drug reactions (ADRs) could be collected separately at any time during the documentation period and up to 90 days after treatment end. Due to the implementation of a new European Union safety directive, collection of ADRs was switched to adverse event (AE) documentation starting from March 2013. AEs documented only in the Roche safety database prior to implementation of the directive were added to the clinical database after SAE reconciliation and prior to database lock.

Due to inconsistencies/incomplete data regarding previous therapy line and tumor assessments, retrospective data cleaning was conducted between June and December 2016. Changes from CR to PR after induction in 5 patients (3 first‐line, 2 relapsed/refractory) as well as changes in the tumor status after maintenance in 34 patients (25 first‐line, 9 relapsed/refractory) impacted the endpoint analysis for PFS and tumor status.

The primary objective was to investigate the therapeutic effectiveness of rituximab maintenance therapy in FL, measured by 2‐year PFS rates in cohorts 1 and 2. Secondary objectives included the estimation of 2‐year OS rates and tumor status at study end. In addition, rituximab‐based treatment schedules in routine clinical practice were recorded and the safety profile of rituximab maintenance therapy described.

Response to therapy was assessed without any prespecified criteria in terms of diagnostic methodology or response assessment time points.

Safety was assessed by ADR/AE analysis as reported by the physicians according to the Medical Dictionary for Regulatory Activities (MedDRA, version 17.0) system organ classes and preferred terms.

### 2.1 Statistical analysis

The study was designed to demonstrate an estimated 2‐year PFS rate of 75% with a precision of ±4.2%, based on an intended total sample size of 500 patients and a share of 20% of censored observations (ie, premature termination without progression). With an intended sample size of 300 treatment‐naïve patients and 200 relapsed/refractory patients, the assumed 2‐year PFS rates were 80% ±5.1% and 70% ± 7.1%, respectively.

All parameters were evaluated in an explorative or descriptive manner. Continuous characteristics are presented by number of observations, mean, standard deviation, minimum, median and maximum, and categorical characteristics with absolute and relative frequencies.

Progression‐free survival (defined as time from first rituximab maintenance administration to first occurrence of progression or death from any cause) and OS (defined as time from first rituximab maintenance administration until death) were assessed by means of Kaplan‐Meier methodology. The Kaplan‐Meier estimates for PFS and OS rates at 2 years are presented with 95% confidence intervals (CIs; using Greenwood's standard error estimate). Subgroup analyses were performed using the same statistical methods. Response and conversion rates associated with rituximab maintenance (from start to end of maintenance therapy) were calculated in a subset of patients who experienced tumor progression within 24 months after first infusion of rituximab, completed maintenance therapy, or had a final tumor assessment 20‐28 months after the onset of maintenance.

## 3 RESULTS

### 3.1 Patients

**Figure 1:**
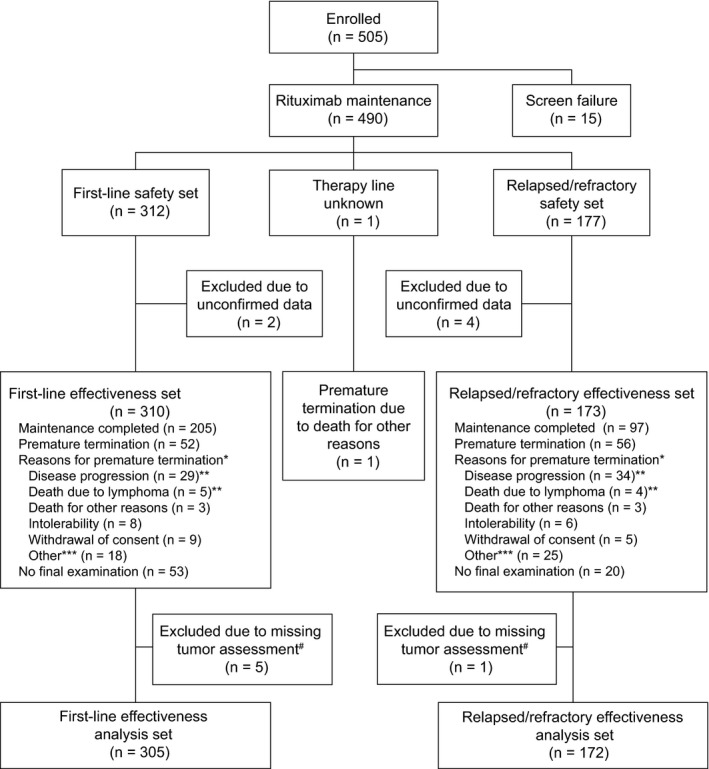
Patient disposition. *Multiple answers possible; **based on safety analysis; ***including comorbidities not primarily associated with FL and not regarded as related to maintenance; ^#^these patients did not have at least one tumor assessment after the first dose of rituximab. FL, follicular lymphoma.

As shown in Figure 1, 490 of 505 patients enrolled initially received at least one infusion of rituximab maintenance therapy. Fifteen patients were excluded due to insufficient documentation and regarded as screening failures. The study population comprised 312 previously untreated patients and 177 patients with relapsed/refractory disease (safety set); for one patient, no therapy line was reported. Two patients were excluded from the first‐line safety set and 4 patients from the relapsed/refractory safety set due to unconfirmed data, leaving 310 and 173 patients, respectively, in each effectiveness set. Five patients of the first‐line effectiveness set and one patient of the relapsed/refractory effectiveness set were excluded from the effectiveness analyses, as these patients did not have at least one tumor assessment after the first dose of rituximab. Baseline patient characteristics of the effectiveness set (overall population, patients with initial Ann‐Arbor stages I/II and patients with FL grade 3B) are provided in Table 1. Median follow‐up time was 21.9 months.

**Table 1:** Patient characteristics

	Overall		Ann‐Arbor stage I+II^a^		FL grade 3B^a^	
	First‐line (n = 310)	Relapsed/refractory (n = 173)	First‐line (n = 67)	Relapsed/refractory (n = 49)	First‐line (n = 12)	Relapsed/refractory (n = 8)
Male sex, n (%)	139 (44.8)	79 (45.7)	30 (44.8)	27 (55.1)	9 (75.9)	2 (25.0)
Median age (range), y	63 (25‐89)	65 (32‐86)	65 (37‐89)	65 (46‐85)	64 (38‐76)	72 (60‐79)
FL grade^a^, n (%)						
1/2/3A	298 (96.1)	165 (95.4)	65 (97.0)	48 (98.0)	‐	‐
3B	12 (3.9)	8 (4.6)	2 (3.0)	1 (2.0)	12 (100.0)	8 (100.0)
Ann‐Arbor stage^a^, n (%)						
I/II	67 (21.6)	49 (28.3)	67 (100.0)	49 (100.0)	2 (16.7)	1 (12.5)
III/IV	243 (78.4)	124 (71.7)	‐	‐	10 (83.3)	7 (87.5)
ECOG^b^ ≥1, n (%)	137 (44.2)	95 (54.9)	29 (43.3)	27 (55.1)	6 (50.0)	7 (87.5)
Time between first diagnosis and initiation of current maintenance therapy^c^, median (range), mo	9.0 (<1.0‐308.5)	60.6 (6.1‐335.3)	9.7 (6.1‐281.3)	68.3 (6.1‐335.3)	6.7 (6.1‐8.8)	60.6 (6.1‐160.4)
Remission status after induction, n (%)						
CR	145 (46.8)	72 (41.6)	38 (56.7)	21 (42.9)	7 (58.3)	1 (12.5)
PR	165 (53.2)	101 (58.4)	29 (43.3)	28 (57.1)	5 (41.7)	7 (87.5)

ECOG, Eastern Cooperative Oncology Group; FL, follicular lymphoma.

a At first diagnosis.

b For two patients with relapsed/refractory FL, no ECOG data were available.

c Date of first diagnosis and/or start date of therapy not always reported or plausible.

### 3.2 Previous and current induction therapies

Relapsed/refractory patients had received up to 6 previous treatments; most (79.2%) were currently treated with second‐line therapy. Median duration of the last previous therapy was 4.4 months (range: 0.0‐51.7), and median time between end of previous therapy and start of current induction was 35.1 months (range: 0.0‐238.2). Previous therapy resulted in CR and PR in 46.2% and 41.6% of patients, respectively.

The median duration of current induction therapy was 4.9 months, with a median of 6 chemoimmunotherapy cycles. At the end of the current induction, 46.8% of the first‐line patients and 41.6% of relapsed/refractory patients achieved CR (PR: 53.2% and 58.4%, respectively) (Table 1).

**Figure 2:**
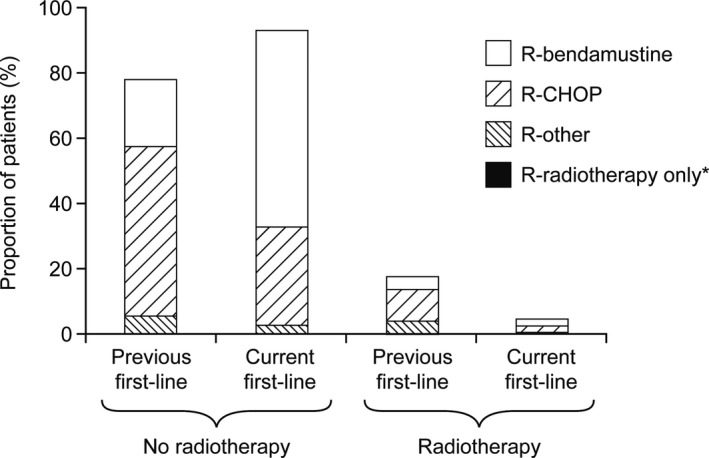
Comparison between previous and current first‐line rituximab‐containing induction therapies. Previous first‐line therapy is shown for relapsed/refractory patients who received the current second‐line treatment following previous rituximab‐containing first‐line induction therapy (n = 100). Current first‐line induction therapy is shown for the cohort of first‐line patients (n = 310). Other regimens included R‐CVP and R‐bendamustine + R‐CHOP. *R‐radiotherapy only in 0.6% of current first‐line patients. CHOP, cyclophosphamide, doxorubicin, vincristine, prednisone; CVP, cyclophosphamide, vincristine, prednisone; R, rituximab.

To compare previous and current first‐line induction therapies, the previous rituximab‐containing induction therapies of second‐line patients were analyzed (while all 310 current first‐line patients received a rituximab‐containing induction, this applied to only 100 of 137 previous first‐line regimes). Figure 2 reveals a shift over time from R‐CHOP to R‐bendamustine as the most frequent first‐line chemoimmunotherapy and a decrease in radiotherapy: R‐bendamustine increased from 21.0% in previous induction to 60.3% in current induction, while R‐CHOP declined from 52.0% to 29.7%. The use of radiotherapy, alone or in combination with chemoimmunotherapy, declined from 18.0% to 4.8%. Rituximab monotherapy was administered to ≤4% of the patients both in previous and current first‐line therapy and to 12.7% (of 173 patients) in current therapy after relapse/refractory disease.

Of the 189 patients who received current first‐line R‐bendamustine (of 305 patients evaluable for effectiveness), 62.4% were female, while only 39.8% of the 98 patients treated with first‐line R‐CHOP were female. In the first‐line R‐bendamustine subgroup, the proportion of patients with stage III/IV at baseline was 77.2% and 1.1% had FL grade 3B, compared with 84.7% and 9.2%, respectively, in the first‐line R‐CHOP group.

### 3.3 Characteristics of rituximab maintenance therapy

The median number of rituximab cycles during maintenance therapy was 12 cycles in first‐line patients (range: 4‐16), with a median duration of 20.8 months. For relapsed/refractory patients, a median of 8 cycles (range: 2‐16) was administered (19.5 months median duration). The median cycle interval was 2.0 months for first‐line patients and 2.9 months for relapsed/refractory patients with a median dose of 375 mg/m^2^ in both cohorts. The median infusion time was 3.0 hours in first‐line patients and 3.5 hours in relapsed/refractory patients.

### 3.4 Effectiveness

**Figure 3:**
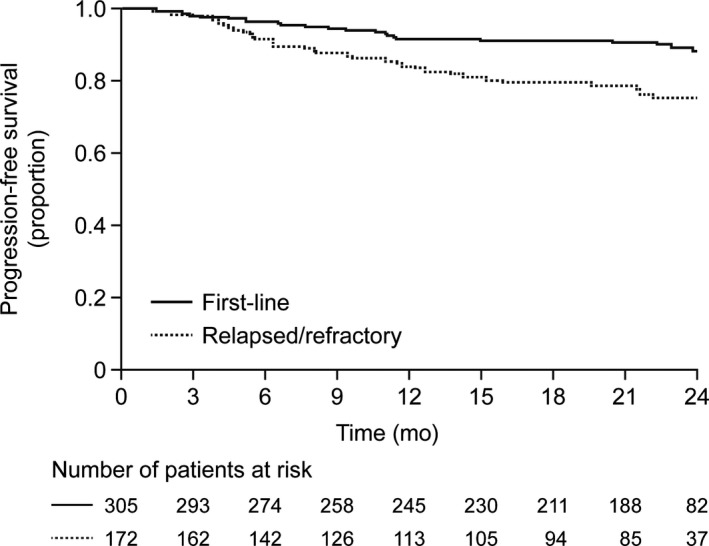
Kaplan‐Meier analysis of progression‐free survival of patients receiving maintenance therapy after first‐line induction (n = 305) and after induction for relapsed/refractory disease (n = 172).

Kaplan‐Meier analysis estimated a 2‐year PFS rate with rituximab maintenance of 88.3% (95% CI: 84.0‐92.6) in first‐line patients and 76.0% in relapsed/refractory patients (95% CI: 68.8‐83.3) (Table 2); median PFS was not reached until end of observation (Figure 3). Further 2‐year PFS estimates by sex, age, stage, FL grade, and induction regime (Table 2) are merely descriptive; no statistical comparisons were performed. Overall 2‐year OS rates were 96.9% (95% CI: 94.7‐99.1) in first‐line patients and 95.4% (95% CI: 91.8‐99.1) in relapsed/refractory patients (Figure S1).

**Table 2:** Two‐year PFS rates of first‐line and relapsed/refractory patients by subgroups

	First‐line			Relapsed/refractory		
	No. of patients	2‐year PFS (%)	95% CI	No. of patients	2‐year PFS (%)	95% CI
Total	305	88.3	84.0‐92.6	172	76.0	68.8‐83.3
Sex						
Male	137	84.5	76.5‐92.6	79	68.6	56.5‐80.6
Female	168	90.9	86.0‐95.8	93	82.3	78.8‐90.8
Age category						
<75 y	257	89.5	85.0‐94.0	147	76.6	69.0‐84.2
≥75 y	48	81.6	68.8‐94.3	25	72.2	49.7‐94.6
Ann‐Arbor stage						
I/II	65	91.6	84.5‐98.7	49	88.1	78.3‐98.0
III/IV	240	87.4	82.3‐92.5	123	71.0	61.8‐80.3
FL grade						
1/2/3A	293	88.5	84.1‐92.9	164	76.0	68.4‐83.4
3B	12	74.1	48.7‐99.5	8	60.0	24.4‐95.6
Induction regime						
R‐bendamustine	189	92.7	88.6‐96.8	113	71.2	61.5‐80.9
R‐CHOP	98	78.8	68.4‐89.1	17	80.4	60.4‐100.0
R‐monotherapy	7	68.6	32.1‐100.0	22	84.4	68.3‐100.0

CHOP, cyclophosphamide, doxorubicin, vincristine, prednisone; CI, confidence interval; PFS, progression‐free survival; R, rituximab.

**Figure 4:**
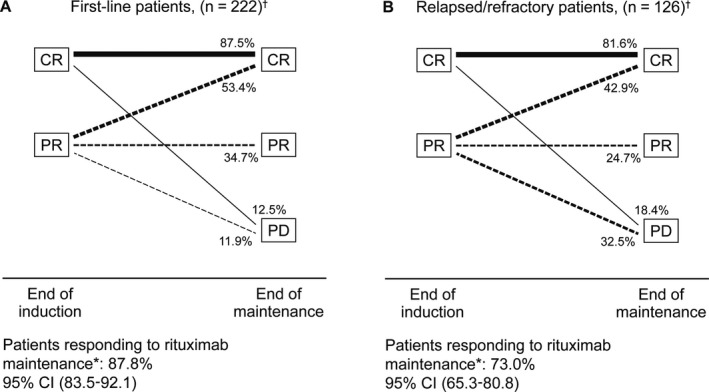
Remission conversion rates from end of induction therapy to end of rituximab maintenance therapy. ^†^Patients with PD ≤24 month after start of maintenance, completed maintenance, or maintenance not completed and last tumor assessment 20‐28 mo after start of maintenance were analyzed (first‐line, n = 222; relapsed/refractory, n = 126); *Percentage of patients with tumor status CR (for patients in CR or PR at end of current induction therapy) and PR (for patients in PR at end of current induction therapy) at the end of rituximab maintenance therapy. CI, confidence interval; CR, complete remission; PD, progressive disease; PR, partial remission.

Figure 4 shows the remission conversion rates from end of induction to end of maintenance in patients with progressive disease ≤24 months after start of maintenance, completed maintenance, or maintenance not completed and last tumor assessment 20‐28 months after start of maintenance (first‐line, n = 222; relapsed/refractory, n = 126). In first‐line therapy, the CR rate increased from 46.8% (95% CI: 40.3‐53.4) after induction to 69.4% (95% CI: 63.3‐75.4) at the end of maintenance, with 53.4% (95% CI: 44.4‐62.4) of the patients with PR at the end of induction having converted to CR; the responder rate at the end of maintenance (patients with CR after postinduction CR/PR, and patients who maintained PR) was 87.8% (95% CI: 83.5‐92.1). Among the patients treated after relapsed or refractory FL, the proportion with CR increased from 38.9% (95% CI: 30.4‐47.4) postinduction to 57.9% (95% CI: 49.3‐66.6) postmaintenance; CR was achieved in 42.9% (95% CI: 31.8‐53.9) of the patients with PR after induction, and the responder rate was 73.0% (95% CI: 65.3‐80.8).

### 3.5 Safety

The switch from ADR to AE documentation limited the AE analysis. Observed AE incidence might have been higher had all AEs been reported from study initiation. AEs and serious AEs (SAEs) were reported in 26.9% and 11.2% of the patients in the first‐line cohort and in 39.0% and 22.0% of the relapsed/refractory cohort. Overall, in 13.5% of the patients ADRs assessed as related to rituximab were observed, with 5.3% serious ADRs (SADRs). The most frequent SAEs and SADRs were leukopenia (reported as SAE in 2.7% of the patients and as SADR in 1.6%), pneumonia (2.0% and 1.6%, respectively), and dyspnea (1.4% and 1.0%, respectively).

Table 3 lists all AEs observed in ≥2.0% of the patients during maintenance therapy. The R‐bendamustine subgroup had higher AE incidence than the R‐CHOP subgroup (33.9% vs 20.0%; SAEs leading to death 3.9% vs 3.3%). The most commonly reported AEs in patients who received R‐bendamustine were leukopenia (10.0%), bacterial infection (6.5%), and neoplasms (5.2%); in patients treated with R‐CHOP, dyspnea was the most common AE (4.2%), followed by leukopenia, neoplasms, and pyrexia (each 3.3%). Notably, infections and infestations occurred in 14.2% of patients in the R‐bendamustine subgroup and in 5.0% of patients in the R‐CHOP subgroup.

**Table 3:** Adverse events during rituximab maintenance observed in ≥2.0% of the patients

MedDRA (Version 17.0)System Organ Class, n (%)Preferred Term	Overall (n = 490)	After induction with R‐bendamustine (n = 310)	After induction with R‐CHOP (n = 120)
Any AE	154 (31.4)	105 (33.9)	24 (20.0)
Any AE related to rituximab (ADR)	66 (13.5)	45 (14.5)	11 (9.2)
SAEs leading to death	16 (3.3)	12 (3.9)	3 (3.3)
Blood and lymphatic system disorders	50 (10.2)	37 (11.9)	5 (4.2)
Leukopenia	42 (8.6)	31 (10.0)	4 (3.3)
Neutropenia	14 (2.9)	11 (3.5)	2 (1.7)
Thrombocytopenia	8 (1.6)	7 (2.3)	‐
Gastrointestinal disorders	32 (6.5)	20 (6.5)	4 (3.3)
Diarrhea	14 (2.9)	10 (3.2)	‐
General disorders and administration site conditions	48 (9.8)	27 (8.7)	10 (8.3)
Fatigue	14 (2.9)	8 (2.6)	2 (1.7)
Pain	18 (3.7)	12 (3.9)	3 (2.5)
Pyrexia	14 (2.9)	8 (2.6)	4 (3.3)
Infections and infestations	60 (12.2)	44 (14.2)	6 (5.0)
Bacterial infection	27 (5.5)	20 (6.5)	1 (0.8)
Pneumonia	10 (2.0)	8 (2.6)	2 (1.7)
Investigations	32 (6.5)	22 (7.1)	5 (4.2)
Blood lactate dehydrogenase increased	8 (1.6)	7 (2.3)	2 (1.7)
Neoplasms benign, malignant, and unspecified (incl. cysts and polyps)	20 (4.1)	16 (5.2)	4 (3.3)
Nervous system disorders	16 (3.2)	12 (3.9)	2 (1.7)
Respiratory, thoracic, and mediastinal disorders	25 (5.1)	17 (5.5)	5 (4.2)
Dyspnea	14 (2.9)	8 (2.6)	5 (4.2)
Skin and subcutaneous tissue disorders	19 (3.9)	11 (3.5)	4 (3.3)

ADR, adverse drug reaction; AE, adverse event; CHOP, cyclophosphamide, doxorubicin, vincristine, prednisone; MedDRA, Medical Dictionary for Regulatory Activities; R, rituximab; SAE, serious adverse event.

In total, 16 patients (3.3% of the overall population) died during the study. For 6 of these patients, multiple reasons were provided as cause of death, without specifying a primary cause. The leading cause of death was disease progression in 9 patients. For 5 patients, the cause of death was not known. Four deaths (0.8% of the overall population) were considered by the investigator to be related to rituximab: 1 patient died of pneumonia; 1 patient died due to respiratory diseases; for 1 patient, pain and herpes zoster infection were documented as causes of death; and 1 case was not further specified.

## 4 DISCUSSION

This noninterventional study mirrored routine clinical practice of FL care during 2009‐2014 in Germany. The 310 patients in first‐line therapy and 173 patients with relapsed/refractory FL were approximately 10 years older than patient populations in large randomized controlled trials (median ages of 63 and 65 years, respectively, compared with 57 years in the PRIMA trial^12^ and 54 years in the EORTC 20981 trial^16^). In addition, our study included more patients with Ann‐Arbor stage I/II (21.6% and 28.3%, respectively, vs 10%^12^ and 0%^16^ in the aforementioned trials). Regarding the administration patterns of maintenance therapy, single infusions of 375 mg/m^2^ rituximab every 2 months in first‐line patients and every 3 months in relapsed/refractory patients were confirmed as widely established dosing schedules.

The observed 2‐year PFS rates of 88.3% in first‐line patients and 76.0% in relapsed/refractory patients were comparable or possibly slightly better than the rates observed in randomized controlled trials,^11^, ^12^, ^16^ despite the differences in patient populations regarding age and disease stage. Our results are also consistent with real‐world data from the noninterventional US National LymphoCare Study (NLCS),^19^ and thus indicate the clinical benefit of 2 years of rituximab maintenance therapy following rituximab‐containing induction.

In addition, PR to CR conversion rates of 53.4% in first‐line patients and 42.9% in relapsed/refractory patients demonstrated that postinduction rituximab may not only maintain, but also improve the quality of remission. However, one must take into account that only patients that roughly match the per protocol population were included in this analysis. In the PRIMA trial, 52% of the patients with PR at randomization converted to CR with rituximab maintenance therapy while only 30% converted in the observation group.^12^ Although no OS benefit could be demonstrated after 6 years of observation,^15^ higher CR rates in first‐line therapy have been suggested to translate into prolonged survival in the long term.^20^

Our study revealed unexpected patterns of treatment in patients with FL grade 3B and stage I/II disease. In contrast to the sustained PFS prolongation shown for patients with FL grades 1‐3A,^12^ no clinical benefit from maintenance could be observed in patients with aggressive B‐cell lymphomas including FL grade 3B.^21^ Therefore, the PRIMA trial excluded patients with FL grade 3B.^12^ However, the current study included 20 patients with FL grade 3B. In addition, about one quarter of the patients recruited in the current trial had stage I or II disease which may be cured by radiotherapy. By contrast, these patients received chemoimmunotherapy followed by rituximab maintenance, a strategy recommended for advanced‐stage FL. Similar observations were made in the NLCS study where 20% of the 541 patients receiving rituximab maintenance were in Ann‐Arbor stage I/II.^19^ In parallel, the use of radiotherapy was remarkably reduced in the current vs the previous first‐line induction therapy. A shift from radiotherapy to early chemotherapy appears to be a global trend.^22^, ^23^

Regarding chemoimmunotherapy regimens, R‐bendamustine replaced R‐CHOP as the most frequently administered first‐line protocol. All first‐line patients in the current trial were treated between 2009 and 2014, and the vast majority of patients treated for first relapse had received their first‐line therapy before 2009. Our results are therefore consistent with the NLCS, which recruited patients from 2004 to 2007: US physicians preferred R‐CHOP (55%), followed by R‐CVP (23%), and R‐fludarabine‐based regimens (16%) as first‐line chemoimmunotherapy. 23 Similar to our findings, females less commonly received anthracyclines.^24^ However, these comparisons were not corrected for covariates.

Based on the uncontrolled, noninterventional character, the study had a number of limitations. Particularly, there may have been significant selection bias regarding the choice of regimen for high‐risk vs low‐risk patients; for example, patients with a higher FL burden/more advanced disease may have been more likely to receive R‐CHOP than R‐bendamustine, and patients with an especially good prognosis may have been preferentially given rituximab monotherapy. Thus, only exploratory assumptions on clinical outcome in regard to FL grade, stage, or treatment patterns can be drawn from our PFS and response analyses.

Our safety results were generally consistent with the known safety profile of rituximab, with similar AE/ADR frequencies observed in first‐line and relapsed/refractory patients—although AE incidence are limited due to a switch in documentation. No unexpected safety signals were reported. Interestingly, toxicity was increased with R‐bendamustine vs R‐CHOP, followed by rituximab maintenance (AE rates 33.9% vs 20.0%). This is in line with first results of the GALLIUM trial in which SAEs were more frequently observed in patients receiving bendamustine than in patients receiving CHOP in conjunction with rituximab or the novel anti‐CD20 antibody obinutuzumab.^25^

In conclusion, our data show favorable 2‐year PFS rates in both treatment‐naïve and relapsed/refractory patients with FL under rituximab maintenance following rituximab‐containing induction in clinical routine. Effectiveness and safety results were in line with data from randomized controlled trials, and tumor conversion rates indicated both maintenance and consolidation of tumor remission.

## CONFLICT OF INTEREST

UD: received speaker's honoraria and research funding from Roche. MR: received speaker's honoraria from Roche. AD: received an honorarium from Roche for congress presentation of the study, and her clinic was compensated for study participation. KK and SK: are employees of Roche Pharma AG. US, SBL, PP, AL, and KJU: declare no conflict of interest.

## References

[1] Armitage JO , Weisenburger DD . New approach to classifying non‐Hodgkin's lymphomas: clinical features of the major histologic subtypes. Non‐Hodgkin's Lymphoma Classification Project. J Clin Oncol. 1998;16:2780‐2795. 9704731 10.1200/JCO.1998.16.8.2780

[2] Morton LM , Wang SS , Devesa SS , Hartge P , Weisenburger DD , Linet MS . Lymphoma incidence patterns by WHO subtype in the United States, 1992‐2001. Blood. 2006;107:265‐276. 16150940 10.1182/blood-2005-06-2508 PMC1895348

[3] Swerdlow SH , Campo E , Pileri SA , et al. The 2016 revision of the World Health Organization classification of lymphoid neoplasms. Blood. 2016;127:2375‐2390. 26980727 10.1182/blood-2016-01-643569 PMC4874220

[4] Wahlin BE , Yri OE , Kimby E , et al. Clinical significance of the WHO grades of follicular lymphoma in a population‐based cohort of 505 patients with long follow‐up times. Br J Haematol. 2012;156:225‐233. 22126847 10.1111/j.1365-2141.2011.08942.x

[5] Dreyling M , Ghielmini M , Rule S , et al. Newly diagnosed and relapsed follicular lymphoma: ESMO Clinical Practice Guidelines for diagnosis, treatment and follow‐up. Ann Oncol. 2016;27:v83‐v90. 27664263 10.1093/annonc/mdw400

[6] Aguiar‐Bujanda D , Blanco‐Sanchez MJ , Hernandez‐Sosa M , Galvan‐Ruiz S , Hernandez‐Sarmiento S . Critical appraisal of rituximab in the maintenance treatment of advanced follicular lymphoma. Cancer Manag Res. 2015;7:319‐330. 26604821 10.2147/CMAR.S69145 PMC4629955

[7] Hiddemann W , Kneba M , Dreyling M , et al. Frontline therapy with rituximab added to the combination of cyclophosphamide, doxorubicin, vincristine, and prednisone (CHOP) significantly improves the outcome for patients with advanced‐stage follicular lymphoma compared with therapy with CHOP alone: results of a prospective randomized study of the German Low‐Grade Lymphoma Study Group. Blood. 2005;106:3725‐3732. 16123223 10.1182/blood-2005-01-0016

[8] Rummel MJ , Niederle N , Maschmeyer G , et al. Bendamustine plus rituximab versus CHOP plus rituximab as first‐line treatment for patients with indolent and mantle‐cell lymphomas: an open‐label, multicentre, randomised, phase 3 non‐inferiority trial. Lancet. 2013;381:1203‐1210. 23433739 10.1016/S0140-6736(12)61763-2

[9] Marcus R , Imrie K , Belch A , et al. CVP chemotherapy plus rituximab compared with CVP as first‐line treatment for advanced follicular lymphoma. Blood. 2005;105:1417‐1423. 15494430 10.1182/blood-2004-08-3175

[10] Martinelli G , Schmitz SF , Utiger U , et al. Long‐term follow‐up of patients with follicular lymphoma receiving single‐agent rituximab at two different schedules in trial SAKK 35/98. J Clin Oncol. 2010;28:4480‐4484. 20697092 10.1200/JCO.2010.28.4786

[11] Hochster H , Weller E , Gascoyne RD , et al. Maintenance rituximab after cyclophosphamide, vincristine, and prednisone prolongs progression‐free survival in advanced indolent lymphoma: results of the randomized phase III ECOG1496 Study. J Clin Oncol. 2009;27:1607‐1614. 19255334 10.1200/JCO.2008.17.1561 PMC2668968

[12] Salles G , Seymour JF , Offner F , et al. Rituximab maintenance for 2 years in patients with high tumour burden follicular lymphoma responding to rituximab plus chemotherapy (PRIMA): a phase 3, randomised controlled trial. Lancet. 2011;377:42‐51. 21176949 10.1016/S0140-6736(10)62175-7

[13] van Oers MH , Van Glabbeke M , Giurgea L , et al. Rituximab maintenance treatment of relapsed/resistant follicular non‐Hodgkin's lymphoma: long‐term outcome of the EORTC 20981 phase III randomized intergroup study. J Clin Oncol. 2010;28:2853‐2858. 20439641 10.1200/JCO.2009.26.5827 PMC2903319

[14] Forstpointner R , Unterhalt M , Dreyling M , et al. Maintenance therapy with rituximab leads to a significant prolongation of response duration after salvage therapy with a combination of rituximab, fludarabine, cyclophosphamide, and mitoxantrone (R‐FCM) in patients with recurring and refractory follicular and mantle cell lymphomas: Results of a prospective randomized study of the German Low Grade Lymphoma Study Group (GLSG). Blood. 2006;108:4003‐4008. 16946304 10.1182/blood-2006-04-016725

[15] Salles GA , Seymour JF , Feugier P , et al. Updated 6 year follow‐up of the prima study confirms the benefit of 2‐year rituximab maintenance in follicular lymphoma patients responding to frontline immunochemotherapy. Blood. 2013;122:509 (abstract).

[16] van Oers MH , Klasa R , Marcus RE , et al. Rituximab maintenance improves clinical outcome of relapsed/resistant follicular non‐Hodgkin lymphoma in patients both with and without rituximab during induction: results of a prospective randomized phase 3 intergroup trial. Blood. 2006;108:3295‐3301. 16873669 10.1182/blood-2006-05-021113

[17] Vidal L , Gafter‐Gvili A , Salles G , et al. Rituximab maintenance improves overall survival of patients with follicular lymphoma‐Individual patient data meta‐analysis. Eur J Cancer. 2017;76:216‐225. 28336303 10.1016/j.ejca.2017.01.021

[18] National Comprehensive Cancer Network . NCCN Clinical Practice Guidelines in Oncology (NCCN Guidelines^®^). Non‐Hodgkin's Lymphomas. Version 4.2014. https://www.nccn.org/about/nhl.pdf. Accessed May 24, 2017.

[19] Nastoupil LJ , Sinha R , Byrtek M , et al. The use and effectiveness of rituximab maintenance in patients with follicular lymphoma diagnosed between 2004 and 2007 in the United States. Cancer. 2014;120:1830‐1837. 24668580 10.1002/cncr.28659 PMC4265986

[20] Bachy E , Brice P , Delarue R , et al. Long‐term follow‐up of patients with newly diagnosed follicular lymphoma in the prerituximab era: effect of response quality on survival–A study from the groupe d'etude des lymphomes de l'adulte. J Clin Oncol. 2010;28:822‐829. 20026809 10.1200/JCO.2009.22.7819

[21] Jaeger U , Trneny M , Melzer H , et al. Rituximab maintenance for patients with aggressive B‐cell lymphoma in first remission: results of the randomized NHL13 trial. Haematologica. 2015;100:955‐963. 25911553 10.3324/haematol.2015.125344 PMC4486230

[22] Vargo JA , Gill BS , Balasubramani GK , Beriwal S . What is the optimal management of early‐stage low‐grade follicular lymphoma in the modern era? Cancer. 2015;121:3325‐3334. 26042364 10.1002/cncr.29491

[23] Friedberg JW , Taylor MD , Cerhan JR , et al. Follicular lymphoma in the United States: first report of the national LymphoCare study. J Clin Oncol. 2009;27:1202‐1208. 19204203 10.1200/JCO.2008.18.1495 PMC2738614

[24] Nabhan C , Zhou X , Day BM , et al. Disease, treatment, and outcome differences between men and women with follicular lymphoma in the United States. Am J Hematol. 2016;91:770‐775. 27124800 10.1002/ajh.24401 PMC5564298

[25] Hiddemann W , Barbui AM , Canales Albendea MA , et al. Immunochemotherapy with obinutuzumab or rituximab in previously untreated follicular lymphoma in the randomised phase III GALLIUM study: analysis by chemotherapy regimen. Hematol Oncol. 2017;35:117‐119 (abstract 107).

