# Supplementary figures and images for: Rituximab maintenance therapy of follicular lymphoma in clinical practice
Source: Cancer Med. 2018 May 15;7(7):2903–12. doi: 10.1002/cam4.1549 (PMC6051161; doi:10.1002/cam4.1549)

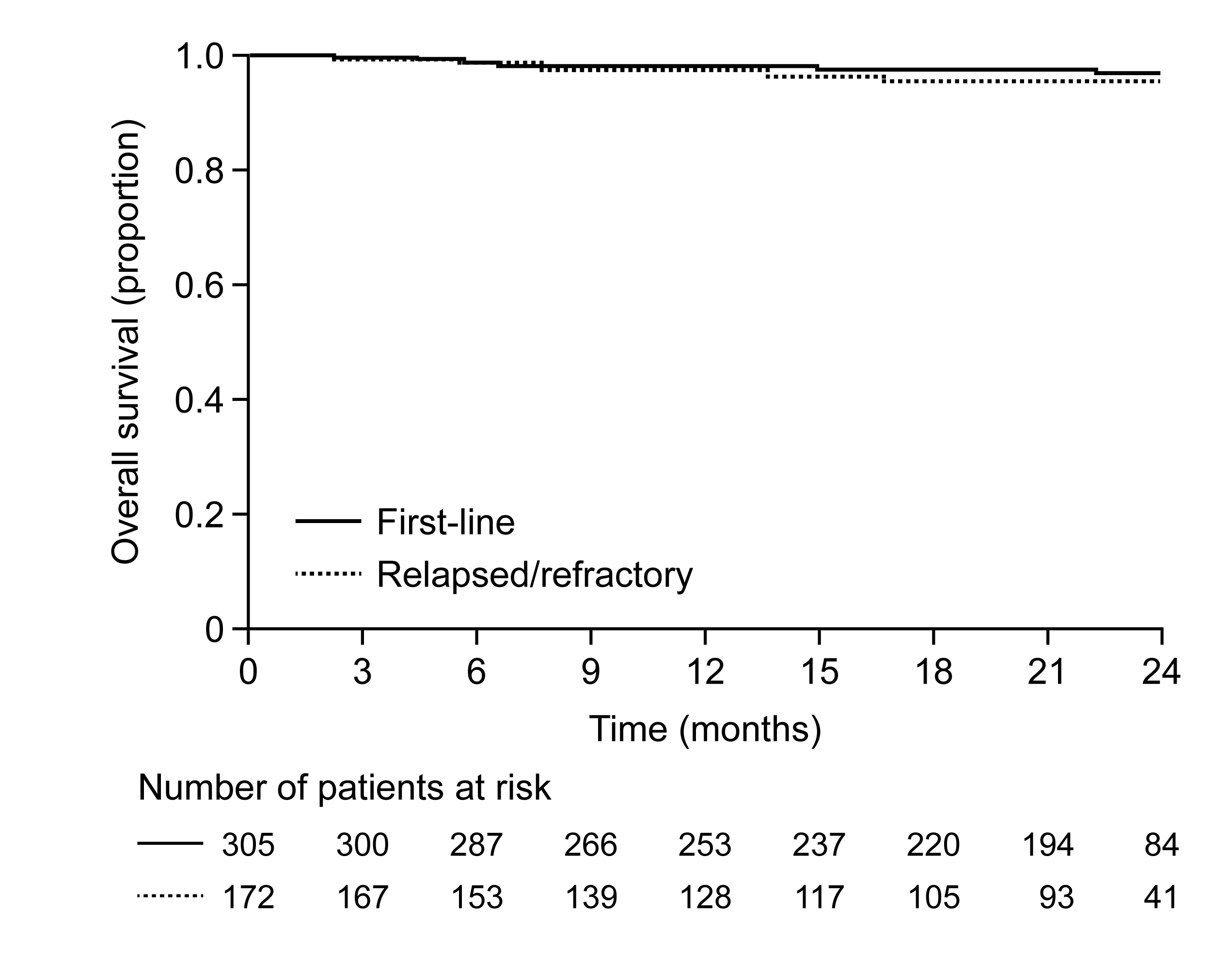

Supplement: Supplementary file 1 [file CAM4-7-2903-s001.tif]
